# Strain-Specific Adaptations of *Streptococcus mitis-oralis* to Serial In Vitro Passage in Daptomycin (DAP): Genotypic and Phenotypic Characteristics

**DOI:** 10.3390/antibiotics9080520

**Published:** 2020-08-15

**Authors:** Nagendra N. Mishra, Truc T. Tran, Cesar A. Arias, Ravin Seepersaud, Paul M. Sullam, Arnold S. Bayer

**Affiliations:** 1 Division of Infectious Diseases, The Lundquist Institute at Harbor-UCLA Medical Center, Torrance, CA 90502, USA; abayer@lundquist.org; 2 David Geffen School of Medicine at the University of California, Los Angeles (UCLA), CA 90024, USA; 3 Center for Antimicrobial Resistance and Microbial Genomics, Division of Infectious Diseases, University of Texas McGovern School of Medicine, Houston, TX 77030, USA; Truc.T.Tran@uth.tmc.edu (T.T.T.); cesar.arias@uth.tmc.edu (C.A.A.); 4 Antimicrobial Resistance Unit and International Center for Microbial Genomics, Universidad El Bosque, Bogota 10121, Colombia; 5 University of California, San Francisco, CA 94143, USA; ravin.seepersaud@icloud.com (R.S.); paul.sullam@ucsf.edu (P.M.S.); 6 VA Medical Center, San Francisco, CA 94121, USA

**Keywords:** *Streptococcus mitis-oralis*, daptomycin resistance

## Abstract

Viridans group streptococci (VGS), especially the *Streptococcus mitis-oralis* subgroup, are pivotal pathogens in a variety of invasive endovascular infections, including “toxic shock” in neutropenic cancer patients and infective endocarditis (IE). Previously, we showed that the serial in vitro passage of *S. mitis-oralis* strains in sublethal daptomycin (DAP) resulted in rapid, high-level and stable DAP-resistance (DAP-R), which is accompanied by distinct changes in several genotypic and phenotypic signatures: (1) the disappearance of two key membrane phospholipids, phosphatidylglycerol (PG) and cardiolipin (CL); (2) increased membrane fluidity; (3) increased positive surface charge; (4) single nucleotide polymorphisms (SNPs) in two loci involved in CL biosynthesis (*pgsA; cdsA*); and (5) DAP hyperaccumulation. The current study examined these same metrics following in vitro serial DAP passages of a separate well-characterized *S. mitis-oralis* bloodstream isolate (SF100). Although some metrics seen in prior DAP post-passage strains were recapitulated with SF100 (e.g., *pgsA* SNPs, enhanced membrane fluidity), we observed the following major differences (comparing the parental versus post-passage variant): (1) no change in PG content; (2) reduced, but not absent, CL, with enhancement in phosphatidic acid (PA) content; (3) an unusual pattern of CL localization; (4) significantly decreased positive surface charge; (5) no difference in DAP accumulation; and (6) no *cdsA* SNPs. Thus, *S. mitis-oralis* strains are not “pre-programmed” phenotypically and/or genotypically to adapt in an identical manner during the evolution of the DAP-R.

## 1. Introduction

Invasive infections caused by the *S. mitis-oralis* subgroup of viridans group streptococci (VGS), especially endovascular syndromes, are increasing in both nosocomial and community settings [1,2,3,4]. For example, *S. mitis-oralis* is the second leading cause of infective endocarditis (IE) in the developing world [1,2,3,4]. In addition, this subgroup is the most common cause of the “toxic *Strep* syndrome” in immunocompromised, neutropenic hosts [5,6,7,8]. Given worldwide trends in penicillin resistance and β-lactam MIC (minimum inhibitory concentration) “creeps” amongst *S*. *mitis-oralis* strains, the proportion of serious infections caused by β-lactam-resistant strains in this VGS subgroup is alarming [8,9,10,11]. Of note, *S*. *mitis*-*oralis* strains have a propensity toward vancomycin-tolerance, further limiting therapeutic options. Collectively, these antibiotic resistance scenarios have rendered daptomycin (DAP) as a plausible option for treating invasive infections with this emerging pathogen. Interestingly, Garcia-de-la-Maria et al. recently reported that >25% of *S. mitis-oralis* clinical strains can rapidly develop high-level and durable DAP-resistance (DAP-R; MICs > 256 µg/mL) following both in vitro and in vivo (experimental IE) exposures to this agent [12]. Such a rapid evolution of high-level DAP-R has not been commonly seen clinically in other Gram-positive endovascular pathogens, including *S. aureus* and enterococci [13,14]. Although DAP-R VGS infections have only recently begun to be reported [12,13,14], the widespread use of DAP (both prophylactically and therapeutically) virtually assures that this scenario will occur more frequently with VGS. In addition, baseline DAP MICs among clinical *S. mitis-oralis* isolates are 2–10-fold higher than all other VGS subgroups [12].

DAP-R in *S. mitis-oralis* is based on unique mechanisms not seen in DAP-R S. *aureus* and enterococci. Thus, prior DAP-R *S. mitis-oralis* strains displayed an essentially complete disappearance of two key cell membrane (CM) phospholipids (PLs), phosphatidylglycerol (PG), and cardiolipin (CL), with the accumulation of phosphatidic acid (PA), the precursor PL in the CL biosynthetic pathway [15]. Of note, whole genome sequencing of several prototypic, in vitro passage-derived, isogenic DAP-S versus DAP-R *S. mitis-oralis* strain-pairs confirmed the presence of non-synonymous mutations within the key gene, *cdsA*, which encodes the first committed enzymatic step in the principal CL synthetic pathway (Figure S1) [15,16,17]. In contrast, in DAP-R *S. mitis-oralis* variants derived from DAP passage within a simulated ex vivo IE model, the above PG-CL disappearance signature was not consistently observed [18], and it was not associated with SNPs in either *pgsA* (also critical PL in the CL biosynthetic pathway) or *cdsA.* Of particular interest, some DAP-R *S. mitis-oralis* strains, particularly those harboring mutations in *cdsA*, show a cell-selective hyperaccumulation of DAP within only certain cocci within a chain, suggesting a form of “altruism” in which one cell subpopulation is ‘protecting’ the overall population [15]. However, this DAP hyperaccumulation phenotype is not uniformly found in all DAP-R strains. Collectively, these findings raised the possibility that the mechanism(s) of DAP-R among distinct *S. mitis-oralis* strains may co-depend on strain-specificities, as well as exposure scenarios (e.g., in vitro versus in simulated vegetations ex vivo versus within in vivo IE lesions).

The objective of this current study was to elucidate the hypothesis that individual *S. mitis-oralis* strains may not be “pre-programmed” in their adaptive pathways in terms of the emergence of DAP-R. Hence, such isolates may evoke and employ unique and multifactorial mechanisms of adaptations to DAP exposure in order to circumvent its bactericidal effects. Therefore, we investigated an additional, well-known S. *mitis-oralis* prototype strain (SF100) [19] isolated from an IE patient following its in vitro passage in DAP [20]. This investigation catalogued key and relevant genotypic and phenotypic perturbations related to the evolution of DAP-R in this strain to understand further the differential adaptation of DAP-R in this strain. This work was presented in-part at ASM Microbe Meeting; New Orleans, LA, USA; June 2017 [20].

## 2. Methods

### 2.1. S. mitis-oralis Strains

The DAP-S parental SF100 S. *mitis-oralis* strain used in this study was identified by MALDI-TOF, and has a DAP MIC of 1 µg/mL [20]). It is a clinical bloodstream isolate from a patient with IE without prior DAP exposures [19]; moreover, it is highly capable of inducing experimental IE [20]. In order to generate DAP-R derivatives, SF100 was subjected to a 20 d serial in vitro passage using concentrations of DAP (20 µg/mL) as described previously [20]. The DAP-R derivative variant was re-passaged in antibiotic-free brain heart infusion (BHI) media for five days to confirm the stability of the DAP-R phenotype [20]. MICs for the serial passage isolates were determined by microbroth dilution, as well as E test assays [15].

### 2.2. Phospholipid (PL) Profiling

Briefly, late stationary phase cells were used to extract the PLs from SF100 and its DAP-R variants, as described previously [15,17,18]. The major PLs (i.e., CL, PG, and PA, as well as a prominent glycolipid [GL] seen in all VGS) were separated by two-dimensional thin-layer chromatography (2D-TLC) (Silica 60 F254 HPTLC plates [Merck]). Identification of all the PL spots on the TLC plates was confirmed by comparison to known standards following exposure to iodine vapors and spraying with CuSO_4_ (100 mg/mL) containing 8% phosphoric acid (v/v) and heated at 180 °C [15,17,18]. The quantification of individual PLs removed directly from TLC plates was performed by digesting with 70% perchloric acid (0.3 mL) at 180 °C for 3 h. The individual PLs were quantified by measuring the optical density at 660 nm using spectrophotometry as described before [15,17,18]. The PL results are expressed as their individual mean proportions (±SD), employing at least three independent experiments performed on different days.

### 2.3. CL Localization

The fluorescent dye 10-*N*-nonyl arcidine orange (NAO) was used to assess the localization of CM anionic PL-rich microdomains by previously described techniques [15]. Briefly, NAO preferentially binds to anionic PL-enriched microdomains (predominantly CL) in the CM. For fluorescence microscopy, *S. mitis-oralis* cells were grown in TSB (Tryptic Soy Broth) to an exponential phase (A600 of approximately 0.3). Then, NAO was added to the growth medium to a final concentration of 1 µM, which did not inhibit the growth of S. *mitis-oralis* strains. Samples were stained for 3.5 h at 37 °C with gentle shaking in the dark (because of light sensitivity). Study strains were washed three times with 0.9% saline post-incubation and immediately immobilized on a coverslip treated with poly-l-lysine (Sigma-Aldrich). Fluorescence imaging and phase-contrast imaging were carried out on an Olympus IX71 microscope with a PlanApo N 100-objective. Green fluorescence from NAO (representing anionic PLs) was detected by using a standard fluorescein isothiocyanate (FITC) filter (excitation at 490 nm and emission at 528 nm). Image acquisition was performed using the SlideBook 5.0 software package. Three independent experiments were performed for each strain on different days.

### 2.4. DAP Binding Assays

Bodipy-DAP (BDP-DAP) was used to measure the interaction of DAP with the parental and DAP-R variants, as outlined before [15,17]. In brief, all study strains were grown in BHI broth at 37 °C, pelleted, and incubated with BDP-DAP (concentrations 4 and 64 μg/mL) in LB (Luria-Bertani) broth supplemented with Ca^2+^ at 50 mg/liter) for 15 min in the dark. After staining, excess BDP-DAP was removed by washing–centrifugation, the pellet was washed twice in LB broth, and then, it was immobilized on a coverslip pretreated with poly-l-lysine (Sigma-Aldrich St. Louis, MO, USA). Bacterial cells were seen with an Olympus IX71 fluorescence microscope equipped with PlanApo N 100× objective. Fluorescence measurements were carried out by using a standard FITC filter. The fluorescence intensity was quantitated and normalized to the protein concentration of the samples to assess the relative amount of BDP-DAP bound to the *S.* *mitis-oralis* isolates, as detailed elsewhere [15,17]. Protein concentrations above were measured by using standard bicinchoninic acid (BCA) protein assay kit (Thermo Scientific, West Hills, CA, USA) as instructed by the manufacturer described before [21,22]

### 2.5. Surface Charge

The relative positive surface charge of SF100 and its DAP-R variants was measured by using the cytochrome C (cyt C) binding assay following a spectrophotometric method [15,17,18]. Briefly, strains were grown in BHI broth, washed with 20 mM MOPS (3-morpholinopropane-1-sulfonic acid) buffer (pH 7.0), and resuspended in the same buffer at OD_578_ = 1.0 Cells were incubated with 0.5 mg/mL cyt C for 10 min, and the amount of cyt C (a highly positively charged molecule) remaining in the supernatant after 15 min exposure was measured spectrophotometrically at OD_530_ nm. The greater the amount of cyt C detected in the supernatant (i.e., more unbound cyt C) equates to a relatively increased positive surface charge [15,17,18]. The quantified data represented the means (±SD) of the amount of cyt C detected in the supernatant from a minimum of three independent experiments.

### 2.6. CM Fluidity

Fluidity was determined by using the fluorescent probe 1,6-diphenyl-1,3,5-hexatriene (DPH; excitation and emission wavelengths are 360 nm and 426 nm, respectively). The protocol of DPH incorporation into the CM has been described in detail previously [15,17,18]. Spectrofluorimetry was used to quantify fluorescence polarization and define the polarization index (PI) value as done before (BioTek SFM 25) [15]. An inverse correlation exists between PI value and fluidity (i.e., a lower PI value equates to a higher extent of CM fluidity) [15,17,18]. A minimum of three independent experiments were conducted for each isolate on different days.

### 2.7. Whole Genome Sequencing

Genomic DNA from SF100 WT and a DAP-resistant variant isolated on day 20 of passage (strain D20) from the VGS strain-set was isolated by cetyltrimethylammonium bromide (CTAB) extraction as described previously [15,20]. Genomic DNA was further purified for sequencing using a PureLink Genome DNA Minikit (Invitrogen Waltham, MA, USA) according to the manufacturer’s instructions [15,20]. PacBio library construction, sequencing, and annotation were conducted by using standard methods [15,20]. The generation of genome assemblies and subsequent identification of single nucleotide polymorphisms (SNPs) and insertion–deletion mutations (indels) were performed as detailed elsewhere [15,20]. All mutations identified by whole genome sequencing were confirmed by PCR and Sanger sequencing.

### 2.8. Statistics

The two-tailed Students *t*-test was utilized for statistical analysis of all quantitative data. *p* values of ≤ 0.05 were considered “significant”.

## 3. Results

### 3.1. Generation of DAP-R S. mitis-oralis Strain SF100

Following a 20 d serial passage in DAP, the DAP MIC of SF100 progressively rose to >256 μg/mL (Table 1) [20]. This phenotype was stable on the passage of this DAP-R variant in antibiotic-free media.

### 3.2. PL Content of DAP-R *S. mitis-oralis* Strains

As noted for other *S. mitis-oralis* strains [15,17,18], 2D-TLC of parental DAP-S strain SF100 revealed four major lipid species: three PLs (PG, CL, and PA) and a large glycolipid (GL) spot. The day 20 (D20) DAP-R passage variant exhibited a significant reduction of CL content as compared to the SF100 parental strain (Table 2). Furthermore, the amount of PG was not altered significantly in the SF100 D20 strain versus the DAP-S parental strain. The quantitative PL assays paralleled the qualitative 2D-TLC plate assays above (Figure S2). Of interest, the current dataset was somewhat distinct from our prior study in which a prototypic DAP-R *S. mitis-oralis* strain showed a virtual absence of both PG and CL in the CM [15].

### 3.3. Distribution of Anionic PL Microdomains

**Figure 1:**
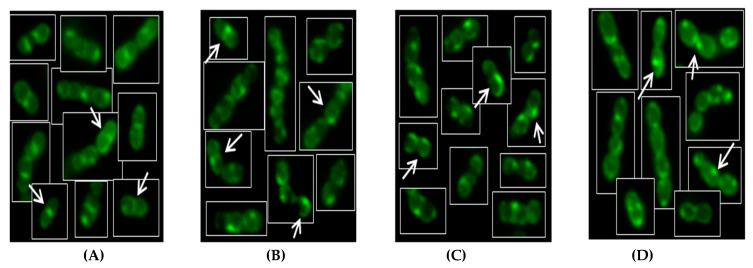
*S. mitis-oralis* DAP-S SF100 and its DAP-R variants at days 10, 15, 20 post-*in vitro* DAP passage. Panel **A**: arrows indicate anionic PL localization at the division septum; Panel **B**–**D**: arrows indicate redistribution of anionic PLs away from division septum in DAP-R derivatives (D10, D15, and D20).

The parental SF100 DAP-S strain (Figure 1A) exhibited anionic PL microdomains mainly located at the septa similar to other DAP-S Gram-positive bacteria [15]. In addition, non-septal discrete fluorescent foci were also observed, which is reminiscent of the ExPortal found in *S. pyogenes* [23,24]. However, in contrast to prior studies [15], septal anionic PL staining was lost in DAP-R derivatives of SF100 (Figure 1B–D); these findings suggested a redistribution of anionic PL microdomains (e.g., CL) away from the septum; this redistribution may well play an important role in DAP-R in *S. mitis-oralis* strains (mirroring similar data in DAP-R *E. faecalis*) [22].

### 3.4. Cell Surface Positive Charge

It has been well-chronicled that many DAP-R *S. aureus,* enterococci, and some prior *S. mitis-oralis* strains exhibit increases in relative positive surface charge versus their respective DAP-S parental strains [15,17,18,21,23]. This DAP-R adaptation has been proposed to generate a “charge-repulsive milieu” that contributes to a reduced ability of calcium–DAP to bind to target CMs to initiate its bactericidal effects. Surprisingly, and in contradistinction to a previous study [15], as SF100 evolved stable DAP-R upon in vitro passage, its surface became substantially less positively charged (Table 3).

### 3.5. DAP CM Binding Profiles

**Figure 2:**
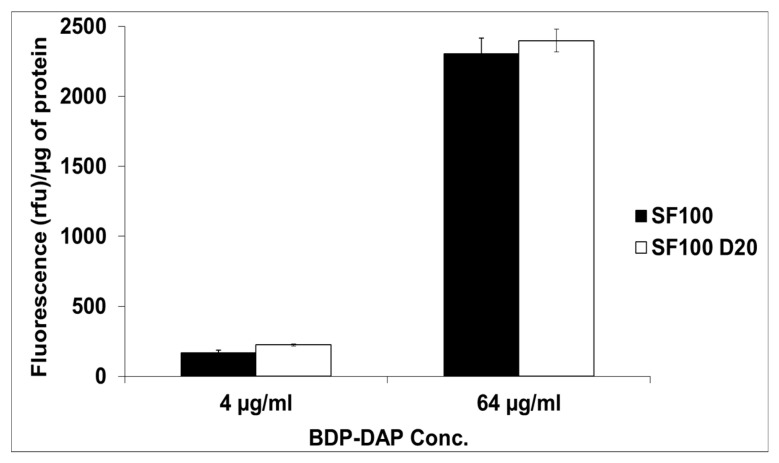
Bodipy-DAP (BDP-DAP) Binding DAP-S SF100 parental versus DAP-R SF100 D20 *S. mitis-oralis*.

As opposed to a prior study [15], in comparing the parental SF100 strain with DAP-R SF100 D20, no quantitative differences in overall DAP binding were detected at either DAP exposure concentration (Figure 2).

### 3.6. CM Fluidity

It is well known that the relative state of the CM “order” has a profound impact on the ability of calcium–DAP and other cationic peptides to interact with the target CM. Interestingly, enhancements in fluidity were, indeed, observed in DAP-R variants; of interest, this phenotype was somewhat delayed during the evolution of DAP-R, becoming evident only in the D20 DAP-R passage variant (Table 3).

### 3.7. Whole-Genome Sequencing (WGS)

WGS of SF100D20 versus the SF100 strain pair revealed a relatively limited number of non-synonymous SNPs and deletions emerging in the DAP-R SF100 D20 passage variant. Of note, these mutations were broadly grouped into those impacting (1) lipid metabolism; (2) cell wall and cell surface homeostasis; or (3) ion transport and the maintenance of CM potential. As underscored above, each of these latter phenotypes are plausibly critical in the interaction of DAP with target CMs [15,17,18,21,22].

As compared with its parent strain, the DAP-R variant, SF100 D20, contains 13 SNPs and 2 deletions, involving a total of 17 ORFs After eliminating genes of unknown function, as well as those with no apparent functional linkage to DAP-R, we identified four candidate genes containing non-synonymous SNPs that could likely be linked to DAP-R (Table 4). Similar to a previous study [17], an SNP in *pgsA* (ORF853) was identified in the SF100 D20 strain; this enzyme catalyzes an important step in CL biosynthesis (Figure S1). In addition, an SNP was found in ORF1433, which is predicted to encode an acetyl–coA-acetyl transferase; this is a key enzyme in the mevalonate pathway, which, in turn, is important for fatty acid metabolism and CM order maintenance, especially under stress conditions (e.g., ‘cold shock’) [25]. ORF1632 (*rpoC*) encodes the β′ subunit of RNA polymerase. Mutations in this protein have been linked to DAP-R [25]. Of interest, RpoBC in *S. aureus* can repress *dlt* expression, the latter being a key regulator of surface charge maintenance [26]. Thus, an SNP in *rpoC* could impact the surface positive charge and potentially perturb calcium–DAP surface binding. SNPs were also found in ORF1590, encoding an alanine dehydrogenase (*ald*) homologue. Of note, mutations in these latter two genes in *S. aureus* have each been associated with cationic antimicrobial peptide resistance [27,28].

## 4. Discussion

The current study with in vitro-derived DAP-R variants emerging from strain SF100 was compelling for a number of reasons. For example, several of the genetic mutations we observed evolving in the SF100 DAP-R variants have been previously linked to DAP-R in other *S. mitis-oralis* (e.g., *pgsA*) [17]. Moreover, other documented mutations we observed were in genes involved in metabolic pathways important for CM biogenesis, integrity, or transmembrane potential maintenance, making them plausible candidates for a role in DAP-R. In addition, several of these genes are important in terms of other metabolic parameters that are likely involved in DAP-R (e.g., fatty acid metabolism; Na^+^/H^+^ antiporters).

Of note, we did not observe non-synonymous mutations in the *cdsA* gene in SF100 D20 DAP-R variants as documented in other DAP-R *S. miti-oralis* [15]. However, it should be emphasized that (1) we did identify SNPs in the *pgsA* gene that catalyze the production of PG, the PL that eventually dimerizes to form CL; and (2) many additional regulatory and metabolic pathways can impact CL synthesis, other than the primary pathway shown in Figure S1 [15]. In this context, WGS of DAP-R in *S. aureus* and *B. subtilis* [25,26] has also identified mutations in a number of genes responsible for PL biosynthesis, including *pgsA*. Thus, it is likely that the mutations identified above by WGS synergize to yield the DAP-R phenotype in SF100.

To assess the phenotypic correlation of mutations in *pgsA* that were found on WGS, 2D TLC and NAO microscopy were performed to quantify proportional PL content, as well as the localization and distribution of anionic PLs, respectively. We recently documented a unique phenotype in other DAP-R *S. mitis-oralis* strains, i.e., an apparent complete disappearance of PG and CL, with the accumulation of the PL precursor, PA [15]. Since negatively charged PG is essential for interaction with, and the oligomerization of DAP within target CMs [29], this phenotype was logically linked to DAP-R. In addition, negatively charged PLs (such as PG and CL) are crucial in DAP interactions within the cell envelope at its major site of activity, the septal division plane. In addition, mutations in *cdsA* and *cls* (leading to alterations in CL content) in DAP-R *S. mitis-oralis* and enterococci have been associated with the diversion and mislocalization of DAP away from the septum [15,22]. Moreover, the combined “microdomains” of PG and CL are known to act as a negatively charged CM “docking site” for DAP and other cationic molecules after initial interaction with and penetration of the CM.

In contrast to these prior investigations [15], we did not observe a “PG–CL disappearance phenotype” among DAP-R variants derived from the in vitro DAP passaging of strain SF100. Of note, DAP-R strains exhibited a similar pattern of PG content versus its DAP-S SF100 parental strain; however, a significant reduction of CL content was noted among the DAP-R variants. It is possible that the mutation in *pgsA* noted above in the DAP-R strain impacted CL content by reducing the amount of PG available for ultimate dimerization into CL.

In addition, NAO staining assays indicated that anionic PL-enriched microdomains were localized at the septum in the DAP-S parental strain as expected. However, in the DAP-R variant, anionic PLs were diverted away from the division septum, likely contributing to DAP-R, as seen in DAP-R enterococci [22].

Another notable feature of prior *S. mitis-oralis* and other DAP-R organisms is increased positive surface charge [15,17,18]. In contrast, in the current study, the net surface charge of the DAP-R variant became less positive during the evolution of DAP-R in vitro. These data underscore that a “charge-repulsive cell surface” is only one of a number of factors that can potentially account for the DAP-R phenotype. In addition, it is theoretically possible that a more negatively charged surface can “trap” more calcium–DAP and prevent it from interfacing with the CM PLs required for its ultimate mechanisms of action.

The DAP binding assays for the DAP-R SF100 variant yielded data with very different outcomes as compared to other DAP-R *S. mitis-oralis* derivatives [15]. Thus, prior studies showed that DAP binding was surprisingly increased overall in DAP-R variants that harbor mutations in *cdsA*, which is associated with the selective hyperaccumulation of DAP in a minority of cocci within individual VGS chains. This supported the notion that in those DAP-R strains, such DAP-hyperaccumulating cells were, in essence, “protecting” the majority of population from exposure to DAP (an example of “altruism”) [15]. In contrast, we found no difference in the quantitative amount of DAP binding when comparing our parent versus DAP-R *S. mitis-oralis* variant.

Alterations in CM fluidity have been linked to DAP-R and cross-resistance to other cationic antimicrobial peptides among other DAP-R Gram-positive pathogens [13,15,17,30,31,32]. It is believed that there exists a fluidity optimum for the interaction of specific cationic antimicrobial peptides (such as calcium-DAP) with target bacterial CMs. The SF100 DAP-R strains exhibited significant increases in CM fluidity versus its DAP-S parental strain, similar to previous observations, suggesting that this biophysical metric may be contributing to DAP-R [15].

## 5. Conclusions

It is likely that *S. mitis-oralis* can develop DAP-R via several mechanisms, as has been shown in studies of DAP-R in *S. aureus* and enterococci [30,31,32]. However, the data presented here, in combination with previous work [15,16,17,33] indicate that altered membrane composition and some other metabolic modifications [34] may be a conserved mechanism for DAP-R. Undoubtedly, specific *S. mitis-oralis* strains are not “pre-programmed” to develop DAP-R by any single pathway.

## Figures and Tables

**Table 1:** List of *S. mitis-oralis* strains and their daptomycin (DAP) MICs.

Strains	Relevant Characteristics	DAP MIC (µg/mL)
SF100	Endocarditis isolate from patient	1
D10	DAP-R derivative produced by in vitro passage	64
D15	DAP-R derivative produced by in vitro passage	>256
D20	DAP-R derivative produced by in vitro passage	>256

**Table 2:** Phospholipids (PL) content of study strains. CL: cardiolipin, PA: phosphatidic acid, PG: phosphatidylglycerol.

Strains	CM PL Composition (% of Total PL [Mean ± SD])		
	PG	CL	PA
SF100	26 ± 6	52 ± 9	21 ± 10
D10	24 ± 6	40 ± 14 *	36 ± 14 *
D15	28 ± 9	32 ± 15 *	40 ± 14 *
D20	34 ± 13	25 ± 11 *	41 ± 17 *

* *p* value < 0.05 vs. SF100 parental strain.

**Table 3:** Positive surface charge and cell membrane (CM) fluidity of *S. mitis-oralis* SF100 and its DAP-R variants at days 10, 15, and 20 post-in vitro DAP passage.

*S. mitis-oralis* Strains	Surface Charge (% of cyt C in Supernatant)	CM Fluidity (PI Value)
SF100	94 ± 13	0.399 ± 0.0
D10	46 ± 17 *	0.350 ± 0.1
D15	75 ± 24	0.388 ± 0.1
D20	62 ± 16 *	0.298 ± 0.0 *

* *p* < 0.05 vs. SF 100 parental.

**Table 4:** Potential candidate genes for mediating DAP-R in *S. mitis-oralis* SF100 D20.

ORF *	NSNP **	AA Change	Name	Biologic Role and Comments
674	G446A	T->I	alanine dehydrogenase (*ald*) homologue	Cellular energy metabolism; SCV (small colony variant) phenotype
1418	C194T	G->E	CDP-diacylglycerol-glycerol-3-phosphate 3-phosphatidyl- transferase (*pgsA*)	CM phospholipid synthesis
1433	G631T	P->T	acetyl-coA acetyl transferase (*thlA*)	mevalonate pathway, integrity of CM order
1632	G1874A	T->I	RNA polymerase β‘ subunit (*rpoC*)	Interacts with *dlt* operon; involved in maintenance of surface charge

* Open reading frame (ORF) in SF100; ** non-synonymous single nucleotide polymorphism.

## Supplementary Materials

The following are available online at https://www.mdpi.com/2079-6382/9/8/520/s1, Figure S1: Major steps in the CL biosynthetic pathways; Figure S2. Major CM PLs of S. mitis-oralis strains. Proportions of PG, CL, and PA in CM of SF100 and its DAP-R derivative SF100 D20 were determined by 2D thin-layer chromatography. GL is a major CM glycolipid found in all VGS.
