# Supplementary material for: Strain-Specific Adaptations of Streptococcus mitis-oralis to Serial In Vitro Passage in Daptomycin (DAP): Genotypic and Phenotypic Characteristics
Source: Antibiotics (Basel). 2020 Aug 15;9(8):520. doi: 10.3390/antibiotics9080520 (PMC7460094; doi:10.3390/antibiotics9080520)
Supplement: Supplementary file 1 [file antibiotics-09-00520-s001.pdf]

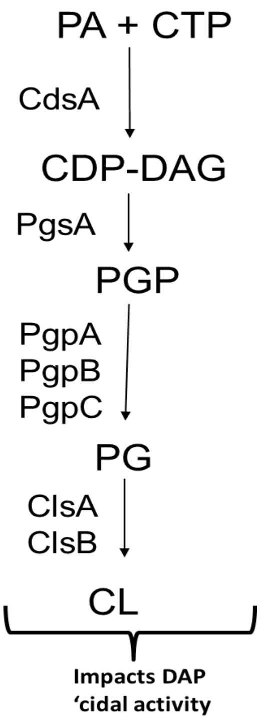

Figure S1. Major steps in the CL biosynthetic pathways.

PA = Phosphatidic acid; CTP = cytidine triphosphate; CDP-DAG = cytidine diphosphate – diacylglycerol; PGP = phosphatidylglycerol-phosphate.

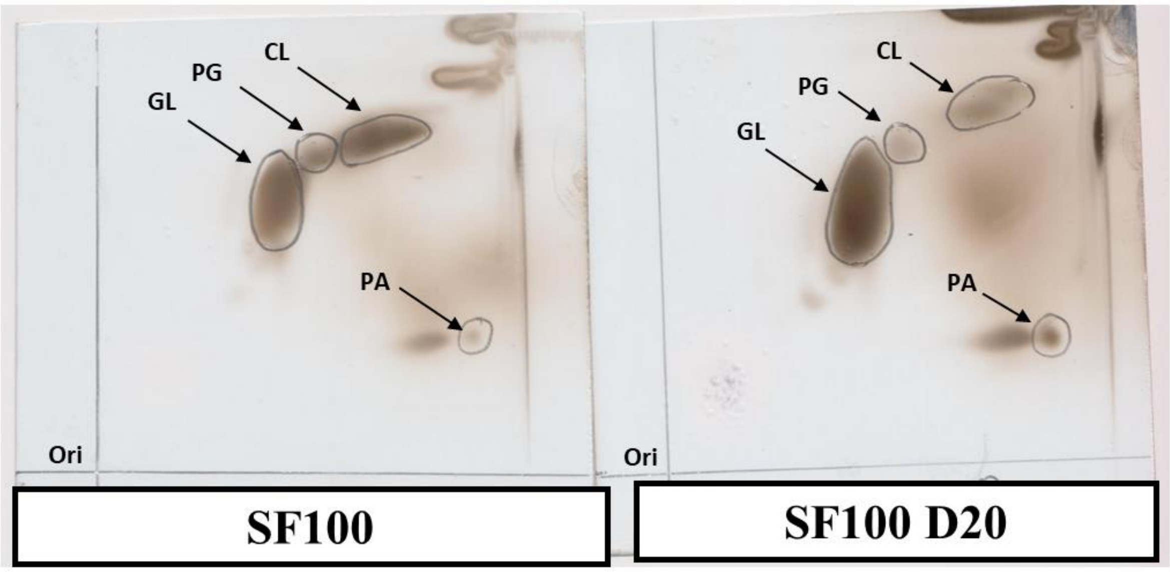

Figure S2. Major CM PLs of S. mitis-oralis strains. Proportions of PG, CL, and PA in CM of SF100 and its DAP-R derivative SF100 D20 were determined by 2D thin-layer chromatography. GL is a major CM glycolipid found in all VGS.
